# An integrated family approach in the practice of adult and child mental health care

**DOI:** 10.3389/fpsyt.2024.1298268

**Published:** 2024-04-15

**Authors:** Hanna Stolper, Karin van Doesum, Majone Steketee

**Affiliations:** ^1^ Departement of Psychology Education and Child Studies, Erasmus University Rotterdam (EUR), Rotterdam, Netherlands; ^2^ Jeugd ggz Dimence Groep, Zwolle, Netherlands; ^3^ Department of Clinical Psychology, Radboud University Nijmegen, Nijmegen, Netherlands; ^4^ Impluz Dimence Groep, Deventer, Netherlands; ^5^ Verwey-Jonker Instituut, Utrecht, Netherlands

**Keywords:** integrated family approach, family focused practice, parent-child relationship, infancy and early childhood, parental mental disorder, mental health care

## Abstract

This paper describes the practice of an integrated family approach to treatment in mental health care in which the focus is on the whole family and treatment is carried out by professionals of adult and child mental health services together. It is presented as an example of a best practice in finding a way to overcome barriers in implementing an integrated family approach in treatment for the benefit of families with a variety of interrelated problems. Even though there is a lot of knowledge about the importance of a family approach in mental health care with specific attention to the patients’ parental role, the children, family relationships, and the social economic context, this is worldwide rarely implemented in the practice of mental health care. Barriers to keep the whole family in mind are identified on different levels: organizational policy, interagency collaboration, professionals, and patients themselves. As a solution, a model of an integrated family approach in mental health care is presented: how it is defined; which domains in the family are targeted; which key elements it contains; what the treatment consists of; and which procedures are followed in practice. A case illustrates how this approach might work in practice.

## Introduction

Although the medical model is still dominant in mental health care, in which mental disorders are viewed as an individual problem and treated as such, the call to consider and include the family context in treatment is becoming louder (1, 2). In this paper we will describe the practice of an integrated family approach in mental health care in which professionals from both the adult and children’s mental health care fields jointly provide treatment for parents and their children with the focus on the whole family.

In the literature, there have been recommendations for considering both parents and children in treatment. The majority of these recommendations are given from the perspective of the practice of adult mental health care and only a few come from the practice of children’s mental health care. Research from the perspective of adult mental health care practice advocate for a Family-Focused Practice (FFP), recommending preventive attention be given to the patient’s family, especially the children (e.g. 3, 4). In response to this, several preventive interventions have been developed for different age groups and are investigated for effect (5–7). Research from the perspective of children’s mental health practices demonstrated that a substantial number of the parents of children who are treated in mental health care suffer from a mental disorder themselves (8–10). This research recommends that the parents of children who receive mental health care should also be screened, and both child and parent should be treated. Since parent and child could adversely affect each other in their reciprocal relationship (11), the need for an integrated treatment of both through close collaboration with a liaison of both adult and children’s mental health services is recommended by several researchers (5, 12). Close collaboration would make multidisciplinary teamwork possible with a focus on the parent and the child, supporting a social and family context for all family members and provide professionals with a space for reflection and careful decision making (13–15).

Despite this knowledge, FFP is rarely practiced in adult mental health care anywhere in the world (2, 16). Likewise, in a case-file study in children’s mental health services, it was found that only in 14,2% of the cases in which the parent and child have concurrent mental disorders, both parent and child received treatment through a professional liaison between adult and children’s mental health services (17). Other research shows that barriers to becoming more family focused are located on different levels: organizational policy; interagency collaboration; professionals; parents and family members. Organizational policy barriers include limited or no resources for professionals to work according to a family focused approach. Issues that they face are: provision of time needed for collaborative work; high workloads; the issue of money, and lack of possibilities for supervision of family interventions (18, 19). The therapeutic point of view within the service can be another barrier for professionals, such as a one-sided orientation on individual mental disorders. Most professionals in adult mental health do not feel comfortable talking to their patients about parenting and the parent-child relationship for various reasons: they do not consider it to be their task; they lack knowledge about parenting and normal child development; they lack the skills to explore this topic, and they are concerned about damaging the therapeutic alliance (18–20). Professionals in child and adolescent mental health care easily overlook mental disorders in the parent because their scope is limited to the symptoms of the child. Therefore, they are less concerned with the functioning of individual adults within the family, including their roles as partners and parents (10, 11). Despite parents’ concerns about the influence of their mental disorder and their style of parenting on the development of their children (21, 22), most of them do not bring up this topic in treatment out of fear of (self) stigma (23), or risk of losing custody of their child (13, 21). Despite this paradox experienced by parents, the majority prefer to discuss this topic and they respond positively to the concept of a treatment approach that is focused on the whole family rather than just on themselves (24).

Based on the aforementioned knowledge, an adult mental health service (AMHS) and a child and adolescent mental health service (CAMHS) in the Netherlands established a partnership in 2012 to offer parents and children treatment with an integrated family approach in which the focus of treatment is on the family as a whole. Integrated treatment offers parents with a diversity of mental disorders and their children the opportunity to receive combined treatment carried out by professionals of adult and children’s mental health services. From this clinical practice, questions emerged from professionals regarding critical targets in treatment to support parents in diminishing the risk of transmitting mental disorders to their children. We investigated the literature to gain insight into the appropriate intervention targets when parents of infants and young children suffer from mental disorders (25). Professionals and patients who participated in these treatments were interviewed to identify the key elements of success in this approach to treatment (26, 27). The findings of this research have directly shaped the practice in the way it has currently developed. In this paper we will describe how the approach is defined; which domains in the family are targeted; which key elements it contains; what the treatment consists of, and which procedures are followed in practice. Furthermore, we will illustrate how this approach might work in practice by presenting a case. Although the integrated treatment in our practice is not limited to a specific age category of the children, in our research we focus on infancy and early childhood (age 0-6). We have narrowed to this group because infants are extremely vulnerable for environmental influences (28, 29), and there is limited preventive and curative support for patients and their young children up to six years (2, 30). The aim of this article is to inspire management and the workforce in mental health care to find a way to overcome barriers and implement an integrated family approach in their treatments for the benefit of parents and their children.

## Context

**Figure 1:**
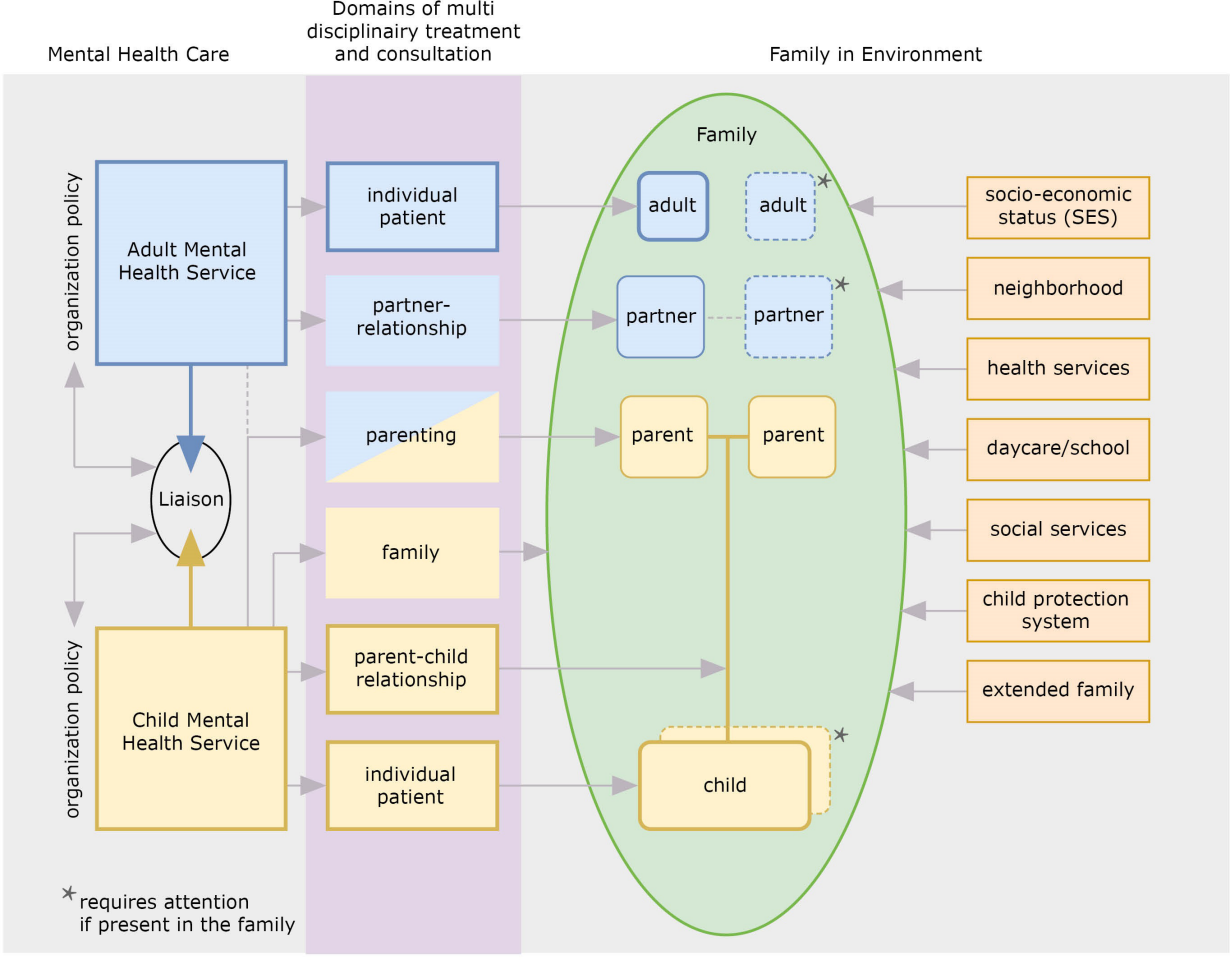
Integrated family approach in mental health care in practice.

Treatment of an integrated family approach offers parents and children a combined treatment carried out by professionals of adult and children’s mental health services. The aim of this approach is “to increase the quality and efficiency of the treatment for parents and their young children, to improve their relationships, and to ameliorate the risk of intergenerational transmission of psychopathology or other adverse outcomes,” (26 p2). This treatment focuses on the current problems in different domains within the whole family. **Figure 1** offers an overview of this approach in which the *family in environment* is the focus of treatment. In the green part are the members of the family presented, which are individuals connected with each other in their reciprocal relationships: the adult with different position or roles; being an individual (with a mental disorder); being a partner in a partner-relationship (if currently present), and being a parent of children. Next there is the other parent (if currently present as part of the family), also seen as being an individual, partner, and parent. Then there is the child (with a mental or relational disorder), and (if currently present) one or more siblings. A family is always part of a society, functioning in a social and economic context which has an influence on how the family functions. The possible, but not complete, aspects of the environment are displayed on the right side of the family. The left side of the figure shows the *adult- and child mental health care services* which are involved with the family. These two mental health care services, with their own financial and organization policies, have implemented a liaison to conduct this approach in their treatments. To be able to integrate their treatments for the different members of the family and their relationships, the involved professionals of the two services participate in a *multi-disciplinary consultation* led by a permanent chairman. To prevent parents and children against the intergenerational transmission of mental disorders treatment should focus on the current problems in *different interrelated domains* within the whole family (25). This includes the mental disorder of the parent(s); the partner relationship; parenthood and family life; and the child’s mental or relational disorder. Furthermore, the mutual relationships, and, especially for infants, the parent-child relationship should be considered (see the purple part of **Figure 1**). The colors blue and yellow refer to the general view of the two services in which domains, and to which members or relationships they offer their treatment. Parenthood and parenting are not self-evident subjects of the treatment at adult mental health service. In general, it depends on the individual view of the professional if parenthood is discussed in treatment. As part of this approach problems in the environmental context of the family, such as the economic situation, should also be examined and to address these problems, a multi-agency approach including social services is needed (25).

The families for whom this integrated treatment is intended are parents and children, with a wide range of types of mental disorders according to the Diagnostic and Statistical Manual of Mental Disorders, fifth edition (DSM-5). These include: personality disorder, depression, bipolar disorder, anxiety, trauma, and neurobiological disorders such as autism and ADHD. With regards to young children, the concern is mainly about relationship disorders, and to a lesser extent neurodevelopmental disorders, trauma, stress, and other disorders according to the Diagnostic Classification of Mental Health and Developmental Disorders of Infancy and Early Childhood (DC:0-5™).

The professionals from AMHS and CAMHS who conduct this integrated treatment are of the following disciplines: psychiatrist, psychotherapist, psychologist (clinical psychologist, general psychologist), group therapist, couples’ therapist, nurse practitioner, professional practicing home treatment.

## Key elements

**Figure 2:**
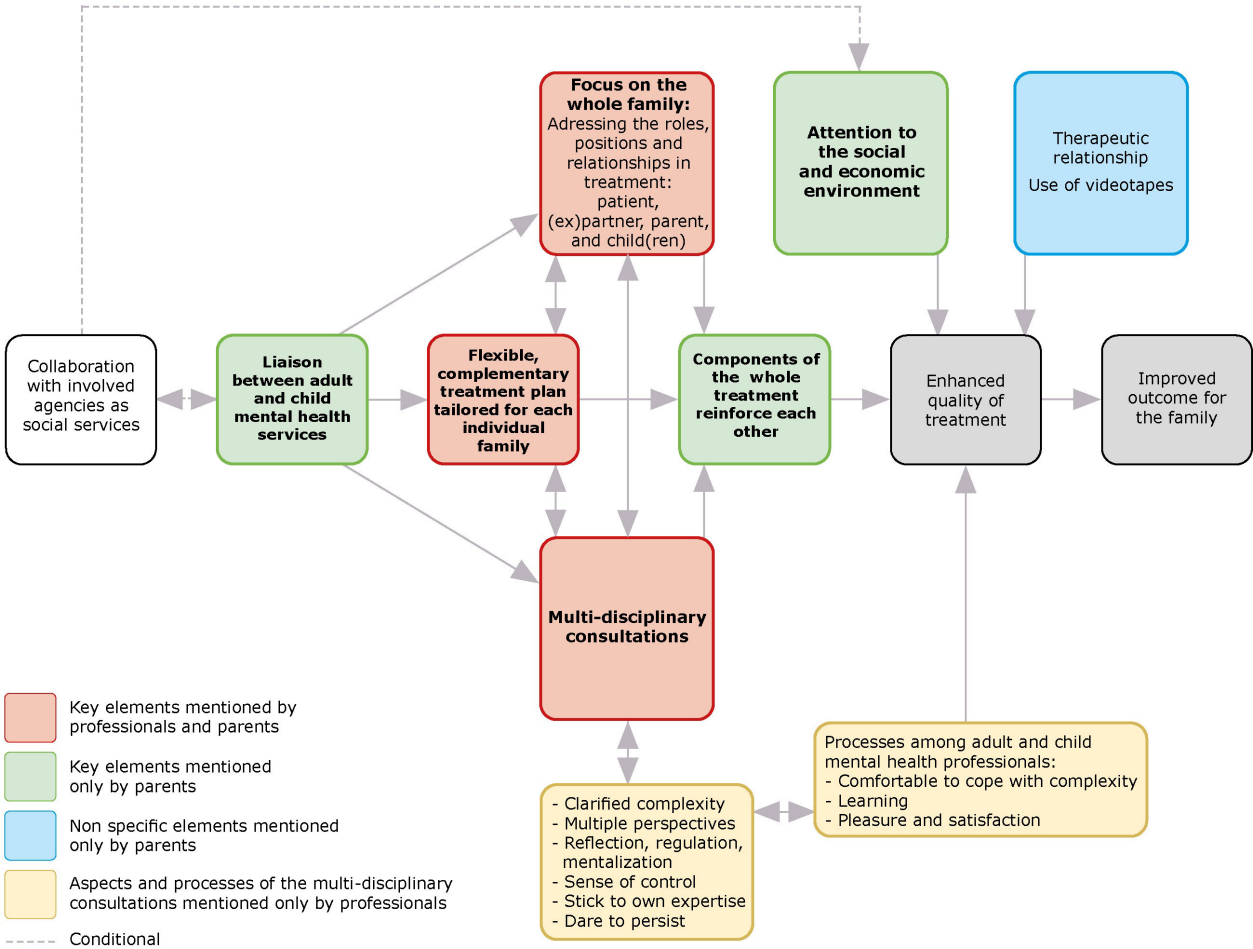
Key elements of success of an integrated family approach in mental health care according to the professionals and parents.

**Figure 2** presents the key elements of success of this integrated treatment according to professionals and patients who have respectively conducted and undertaken the treatment (26, 27). There are six key elements mentioned: 1. focus on the whole family; 2. flexible and complementary treatment plan tailored for each individual family; 3. multi-disciplinary consultations; 4. components of the whole treatment reinforced each other; 5. the liaison between AMHS and CAMHS, and 6. attention to the social and economic environment. In addition to these key elements, parents mentioned two non-specific elements which, in their view, contributed to treatment success: 1. the therapeutic relationship, and 2. the use of videotapes.

The integrated treatment utilizes a *flexible complementary treatment plan* tailored to the needs of family members with the interest of *the whole family*. In doing so, the distinct problems related to the roles and positions within a family are addressed. Professionals from both adult and child outpatient services carry out their treatments according to their own expertise, in parallel or in an integrated manner, but always in close cooperation. If necessary, they carry out a treatment together, for instance, if a parent-infant group is provided.

There is not a specific treatment protocol. Tailoring the treatment to the specific issues within the family, requires a flexible approach and attitude in treatment, and commitment to the interest of all family members (26). **Table 1** shows possible treatment components in our practice targeting the family functioning in different domains. This list gives an impression of which treatments we can provide and combine.

**Table 1:** Possible treatments in different domains which could combined according to an integrated family approach in AMHS and CAMHS.

Domain	Disorder	Treatment
Parental mental disorder	Personality disorders	Individual and group treatment based on Mentalization Based Treatment (MBT) (31) Schema Focused Therapy (SFT) (32) Guideline Informed Treatment for Personality Disorders (GIT-PD) (33) Systems Training for Emotional Predictability and Problem Solving (STEPPS) (34) Pharmacotherapy and physical health check
	Depression and anxiety disorders	Cognitive Behavioral Therapy (CBT) (35) Schema Focused Therapy (SFT) (32) Short-term Psychodynamic Supportive Psychotherapy (SPSP) (36) Pharmacotherapy and physical health check
	Post-traumatic stress disorders	Eye Movement Desensitization and Reprocessing (EMDR) (37) Imaginary Exposure
	Neurodevelopmental disorders: autism spectrum disorders (ASD) and ADHD	Psychoeducation Cognitive Behavioral Therapy (CBT) (35) Competitive Memory Training. (COMET) (38) Mindfulness (39) Psychomotor (family-) therapy (PMT) (40) Art therapy (41), Music therapy (42) Partner group Pharmacotherapy and physical health check
Partner relationship		Partner-relation therapy (43)
Parenthood and family life		Home treatment (Intensive Family Treatment) (44) Parent counseling with both (ex/co) parents Family therapy sessions with members of the extended family and other important relationships
Parent-infant relationship		Assessment of the parent-infant relationship according to the Emotional Availability Scales (EAS) (45) Assessment of atypical parental behavior according to the Atypical Maternal Behavior Instrument for Assessment and Classification (AMBIANCE) (46) Attachment based interventions: Parent-child psychotherapy, if possible, with both parents and infant (47) Modified Interaction Guidance (MIG) (48) Parent- infant group therapy Circle of security intervention (49) Parent-baby intervention (50)
Child (age 0-6)	Post-traumatic stress disorder	Eye Movement Desensitization and Reprocessing (EMDR) (37) Storytelling according to Lovett in the context of the current attachment relationship (51)
	Neurodevelopmental disorders: autism spectrum disorders (ASD) and ADHD	Autism Diagnostic Observation Schedule (ADOS) (52) Psychoeducation Pivotal Response Treatment (PRT) Home treatment (Intensive Family Treatment) (44) Parent counseling with both (ex/co) parents Pharmacotherapy and physical health check
	Other mental disorders	Home treatment (Intensive Family Treatment) (44) Parent counseling with both (ex/co) parents Parent-child psychotherapy, if possible, with both parents and infant (47)
Environment		Collaborating with social services with the aim to reduce the impact of environmental risks (e.g., housing, financial, poverty, criminality, stress) and to enhance social support (extended family, friendships), and if necessary, make provision for alternative care Collaborating with Child Welfare Services for safety in the family Collaborating with daycare, school and health organizations

Although a shared therapeutic view between all involved professionals is desirable, this is in practice not always realistic. In our practice the professionals from AMHS are working in different teams treating different groups of patients with different methods. For instance, patients with a personality disorder are treated with mentalization based treatment (MBT), and patients with anxiety and depression are treated with cognitive behavioral treatment (CBT). Professionals who are treating infants (age 0-6) are guided by an infant and early childhood mental health (IECMH) vision. The essential focus of IECMH is on promoting the quality of the parent-child relationship as the most important context from which development occurs at a stage in which the foundation for mental health is being formed. Treatment focused on the system of the parent-child relationship in which both the child and the parents have their contributions in shaping their patterns (53). Despite differences in therapeutic concepts and methods, professionals are able to find common ground in *focusing on the family as a whole* and *functioning in a social community*.

The involved professionals are employed by and primarily work for their service of AMHS or CAMHS. For the families in their caseload in which both the parent and the young child are in treatment, they participate in a regularly online *multidisciplinary consultation*, with a fixed group of professionals from both services attending each meeting and led by a chairman. In this way expertise from AMHS and CAMHS is brought together for the benefit of the whole family. In this multidisciplinary consultation professionals jointly determine which targets of intervention should be prioritized to initiate positive cascade effects. In addition, they determine in which sequence, time schedule, and with which professionals (see **Figure 1**) the intervention will take place (25). Naturally, all of this is done with the informed consent of the parents who do not participate in the multi-disciplinary consultations. The treatment is regularly evaluated and adjusted where appropriate, according to need and urgency.

Professionals experience the multidisciplinary consultation as an important means of support in carrying out treatment, by being better able to cope with the complexity of problems in families (26). It provides them with clarity on the complexity of the problems. It offers them different perspectives on the family. Through reflecting and mentalizing together, they are better able to regulate intense emotions provoked by the complexity of the problems, resulting in a sense of control and being better able to stick to their own expertise, daring more in treatment and persisting longer by treating in collaboration instead of alone. In addition, they experience that conducting this integrated treatment provides personal learning and brings them greater satisfaction. Importantly, parents report that *processes of different treatments can reinforce each other.* Both professionals and parents indicate that all of these elements of an integrated treatment contribute to *enhanced quality of treatment* and an *improved outcome for the family*.

Stressors in the environmental domain (e.g., housing problems, low socio-economic status), are also mentioned as an important target of intervention to prevent families from experiencing intergenerational transmission of mental disorders (25). Diminishing the stress and social consequences of environmental risk factors and enhancing protective factors (e.g., social support) is part of social work intervention. Hence, a multi-agency approach and collaboration between mental health services, social services, child services are a necessity in this integrated family approach. In our practice professionals from these services, mostly social workers, can regularly join multidisciplinary consultations if the parents give their consent.

To enable an integrated family approach between AMHS and CAMHS, these services are engaged *in a liaison* facilitating the integrated treatment. Mutual commitments are made, for instance, regarding multi-disciplinary consultation, joint treatment, financing, and how responsibilities are allocated. With respect to the latter, a commitment is made that the responsibility for the treatment of the adult patient and the child respectively remains with the responsible professional from AMHS and CAMHS. Because the treatment is conducted by different mental health services, there are always at least two case files, one about the treatment of the parent and one of the child. If further family members are treated there will be a case file for each family member. A shared case file about the whole treatment is impossible due to the rules of the two organizations, but sharing information is allowed with consent of the parent(s).

Referrals for an integrated treatment are made from both AMHS and CAMHS, or from social services, general practitioners, child protection, and hospitals. Indications for referral are: if there are concerns about the mental health of the parent(s) and its impact on parenthood, and concerns about the emotional development and mental health of the child(ren).

To lower the barrier for both the professional and the parent to make use of an integrated treatment, and to increase the likelihood of a successful referral to the other service, there is the option to ask for a mutual consultation. A CAMHS professional can join in a conversation to talk about the patients’ concerns about the children. Vice versa an AMHS professional can join in a session to talk about the concerns of the parents’ mental health and possibilities for treatment.

For professionals there is the possibility to be trained in an integrated family approach The training includes the key elements and focuses on the underpinning theory and the need of the focus on the whole family and to tailor treatment to the individual family. These include, for instance, the processes of and intergenerational transmission of mental disorders (54), risk and protective factors (25), the consequences of adverse child experiences (ACEs) (55), infancy and the attachment theory.

## Case

### The family

**Figure 3:**
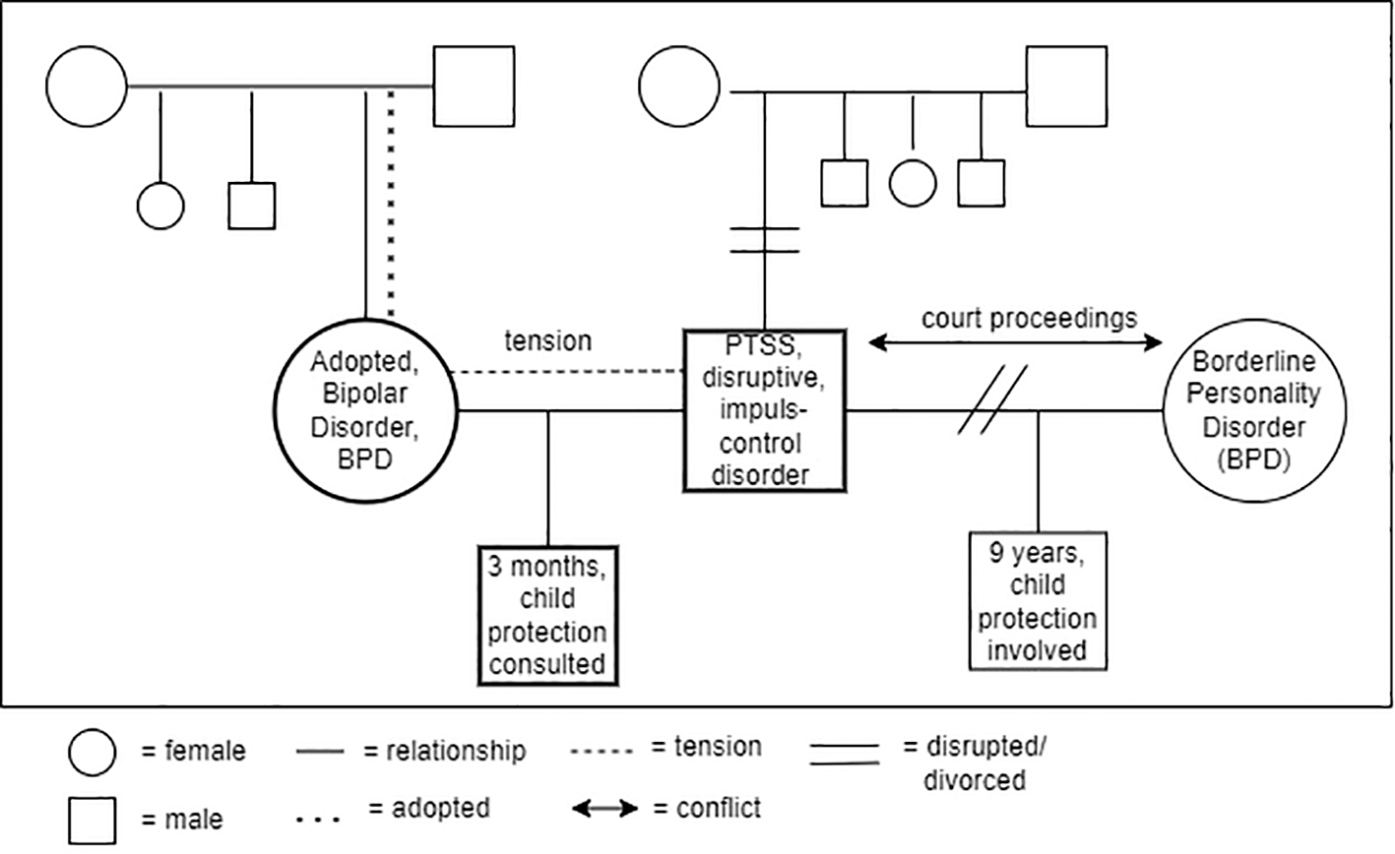
Genogram of the family.

*The mother* is a woman with bipolar, borderline personality disorder (BPS), and at risk for eating disorders. She was in treatment at AMHS for several years. She had a relationship with a man, a machinist, and they had a child (see **Figure 3**). During pregnancy, which was unplanned, she went through a manic episode and was optimistic about parenthood of the child. After delivery, she fell into a severe depression which necessitated admission to a psychiatric hospital on two occasions. The psychiatrist referred the child to CAMHS to establish an integrated treatment because of concerns about the development of the mother-child relationship. The mother believed that she could never take care of her son, and she did not feel like he was her baby. She felt guilty about his existence, and she wanted to find out what would be possible for her in parenthood.

*The father* was in treatment for PTSD at another mental health service. The baby was his second child. He had a son (nine years old) from a previous relationship, who was living with his mother, but spent a few days with his father every week. The father experienced a lot of stress in this parental role because of an ongoing conflict with the mother of his older son that led to him losing his temper a number of times. The child protection service was involved and there were several court proceedings. The father had lost contact with his own family and had no other support network. The father had debts and was not permitted to leave work to take care of his baby son. For that reason, *the baby* (three months old) lived with his grandparents (adoption parents of mother). He was assessed as having light motor delay and sometimes screamed without a visible cause. When he was 2.5 months his leg was broken due to rough handling by the father when changing a nappy. For that reason, child protection services was consulted. The father explained that he was so stressed at that time that he could not control himself. He was shocked and traumatized by this incident and did not dare to care for the baby anymore. The parents of the baby experienced a lot of tension in their *partner relationship*.

*The grandparents* took the baby to the hospital where the mother was being treated every day to stimulate the bond between mother and son.

*The services involved*: two adult mental health services and one child mental health service; social services; supportive guidance with a practical focus at home; child protection services (assessment of safety); financial monitoring and debt assistance.

### The treatment

On the *individual domain* the mother was initially in hospital but after a few weeks, treatment was continued at the outpatient clinic and consisted of medication and weekly visits from a nurse practitioner. After stabilization she began psychotherapy. When the father finished the EMDR, he was referred to schema focused therapy for better emotion and behavioral regulation, but he had to wait several months before he could start. The problems in the *partner relationship* were addressed by therapy at AMHS.

The mother visited her baby once a week at her parents’ house and they brought the baby to the parents for sessions at CAMHS. The latter started with a parent-child observation, which was filmed and reviewed with them. It continued with *parent-child treatment* and was followed by participation in the parent-child group. The focus in the treatment at CAMHS was to help the parents mentalize about their baby and themselves by observing, exchanging thoughts and expressing feelings. If appropriate, some psychological education was provided. For instance, during the observation the baby displayed staring behavior a few times and they disclosed that he did this quite frequently at home. They found this miraculous. By explaining to them that he was disconnected at that time and making them aware of the importance of connection for the baby, they were able to help him to restore the connection with them. In the 12-week parent-child group (AMHS and CAMHS), the father was involved for several sessions. It was observed that the child was avoiding the mother. After exploring he did not return to his mother. The mother in turn let him go and would not impose herself on him, because of her own experience in childhood in which she experienced her adoptive parents as intrusive. She learned the importance of being present, to let him know that she is there for him. The father learned to reconnect with his son and overcame his trauma-related anxiety. The treatment at CAMHS was focused on helping the parents to be sensitive to their baby and confident about their parenthood.

The *grandparents* were involved in treatment to explore their needs and their willingness and confidence in transferring a part of the care for the child to his mother.

*Multi-disciplinary consultations*: The involved professionals from AMHS and CAMHS met regularly and mentalized about the needs of the whole family, the timing of interventions tailored to this specific family, and the complex relationships within it; for instance, the important protective factor of the grandparents and the complex feelings of ambivalence the mother bore towards them. On the one hand she wished to be more independent and on the other hand was the knowledge of her dependence on them for the care for her child. Also, the safety issues were discussed and monitored. The nurse practitioner was in touch with both the social services and the supportive guidance at home.

After more than a year the integrated treatment was completed, although the individual treatments of the parents continued. Because of the continuing stress experienced by the father which led to aggressive verbal expressions, the mother divorced from the father. She lived with her son in her own apartment and was able to take care of him two days and one night a week and the grandparents took the other part of the week. The father visited the son regularly. He was satisfied with the treatment and felt no need for further help to foster the bond with his son. During the evaluation with the mother and her son, he sat on her lap and molded himself to her body. After a while he went off to explore the room. She said, “I really feel like his mother now…. He is my child”.

## Discussion

In this paper we have outlined the use of an integrated family approach to mental health care. Professionals of both adult and child mental health care services provide the family with a flexible and tailored treatment with different components targeting the complexity of problems in the different domains (adult, child, relationships). The involved professionals meet regularly in a multidisciplinary consultation. If professionals from other social or child services are involved in helping the family, they may join the multidisciplinary consultations with parental consent.

It seems that with this practice a number of barriers mentioned in the literature to become more family focused are overcome. Organizational barriers have been overcome by a liaison between AMHS and CAMHS (5, 12), and professionals are facilitated in collaborative treatment by, for instance, joining the multidisciplinary consultations. These consultations give them space for reflection and careful decision making (13–15). Furthermore, joint treatment as well as interagency collaboration with social and child services to address problems in the environment is made possible.

The hesitation of AMHS professionals to talk with their patients about parenthood and the children is not easy to overcome in a huge mental health organization. Of course, there are still professionals who do not count it as their task, despite the law in the Netherlands, to assess the safety of the children in the family (56). Nevertheless, the awareness of the importance of the parental role for their patients and the possible consequences of an unhealthy family life for the development of the children is growing. The possibility to adopt an integrated family approach in their treatment is a supportive factor. After all, what is the point of attention to the children if you cannot address the concerns you might hear. The possibility to ask a colleague from CAMHS to join in a consultation to talk about the parents’ concerns of the children is also helpful. The barrier of professionals at CAMHS, to focus only on the child and overlook the presence of parental mental dysregulation seems to be lowered by the possibility for consultation with a colleague from AMHS. Moreover, professionals are allowed to follow a training program which enhances their knowledge, their sensitivity for the context of the family, and their understanding of the social and economic environment. It also provides them with the space to develop skills to talk about family issues (13, 57, 58).

The mentioned barrier experienced by the patient, the fear of stigma and losing the custody of the child (13, 21, 23), requires a sensitive and brave professional who is able to discuss this in a supportive manner. The possibility to ask a colleague for consultation is useful to overcome barriers that the patients/parents face in considering treatment for the child or for themselves. Despite all of these possibilities to become more family focused and help families to prevent the intergenerational transmission of problems and disorders, waiting lists remain a continuing threat to an integrated family approach in treatment.

The key success factors are the multidisciplinary consultations between all professionals involved. This allows them to focus on the whole family and implement a flexible treatment plan tailored to the individual family’s capabilities and situation. The different treatment components can reinforce each other, improving the quality of treatment and the outcome for the family.

An integrated family approach can be viewed as Family-Focused Practice (FFP), when the latter is defined as a continuum from low to high categories of family-focused activities in which the unit of care is the entire family (3). However, most of the research about FFP advocates for the prevention of problems in children of parents with mental disorders and therefore promotes preventive activities in AMHS. The Family Model (TFM) (58, 59) provides a framework to assist professionals in taking a family focused perspective in treatment and collaborate with other services. A visual tool shows the reciprocal relationships within the family, the context of involved services, risk factors, protective factors, and the social and cultural context. TFM could be used in both AMHS and CAMHS because it aims to broaden the scope from an individual to a family approach and promote a collaboration between adult and child mental health services. However, TFM is mainly used in AMHS to talk with patients who are also parents (58). An integrated treatment can be seen as a practical elaboration of TFM for families in mental health care services with a complexity of problems. This elaboration means that there are problems in different domains (e.g., parent, child, family, environment), for which several services are involved in the treatment with the aim to help the family.

### Learned lessons

- As long as an integrated treatment it is not part of the core of mental health care it remains an ongoing process of sharing knowledge with professionals and training them, and in addition, keeping it on management’s agenda.
- The value of an integrated treatment seems to be the acknowledgement of the interrelatedness of problems in different domains, and that the probability of change requires an integrated approach to these problems.
- The quality of the multi-disciplinary consultations is not warranted just by engaging with professionals and talking about the treatment of the family.
- Only if the multi-disciplinary consultation succeeds in providing the involved professionals with added value in their therapeutic work, will they be motivated to practice a family approach to their treatments.
- A chairman is needed who is familiar with an integrated family approach and stimulates the mentalizing processes among professionals about the families and themselves.
- An integrated treatment contributes to a proactive organization that develops, learns from others and continues to innovate.
- It is an illusion to think that this approach is the solution to all families with complex problems. Humility will be a part of our professional attitude with all patients, but especially in working with this specific group of patients.
- A separate, permanent, and fixed specialist team that treats these families with a complexity of problems in different domain would be an alternative, but maintains fragmentation in mental health care services.
- Administrative staff support is crucial for organizing multidisciplinary consultations across organizations.

### Strength and limitations

A strength of this case study is the evidence- and practice-based foundation and its contribution to clinical practice. In particular, there is a lot of research about the barriers to family focused practice in mental health care, but barely any research presenting best practices to overcome these barriers. Furthermore, this practice offers an opportunity to reach the youngest and most vulnerable family members, of whom mental health is often overlooked by professionals in health services. In addition, although there is a wide variety in the phenomenology of mental disorders in clinical practice most research of parents of mental disorders and children focuses on parents with a specific classification according to the DSM-5. The heterogeneity of the phenomenology of parental mental disorders and the circumstances in which families are living, implies that a “one size fits all” approach is not appropriate. Within treatment of an integrated family approach different combinations of evidence and practice-based treatment are offered for a wide range of mental disorders and relationship disorders. Limitations of the included research can be found in the papers in which this research is presented (25–27).

## Conclusion and recommendations

An integrated family approach to the treatment of patients in mental health care can be helpful to families with a variety of interrelated problems in different domains. It offers professionals and mental health organizations a model of the key elements of this approach and an overview of the domains to intervene in when helping parents to prevent the intergenerational transmission of problems and mental disorders. Therefore, we recommend that mental health organizations actively facilitate professionals in adopting an integrated family approach. This approach will enable and encourage them to include the whole family in their treatments and prevent families from receiving fragmented care. By considering and treating the individual mental disorders of their patients as being part of a comprehensive context of family and society, they can further increase the value for both the families and for their organization. This will result in not only a curative contribution, but also a preventive one, helping to protect the next generation from becoming the patients of tomorrow.

## Data availability statement

The raw data supporting the conclusions of this article will be made available by the authors, without undue reservation.

## Ethics statement

The studies involving humans were approved by Medical Ethics Review Board at the University Medical Centre of Utrecht in the Netherlands (18-186/C). The studies were conducted in accordance with the local legislation and institutional requirements. Written informed consent for participation in this study was provided by the participants' legal guardians/next of kin. Written informed consent was obtained from the individual(s), and minor(s)' legal guardian/next of kin, for the publication of any potentially identifiable images or data included in this article.

## Author contributions

HS: Conceptualization, Data curation, Formal analysis, Investigation, Methodology, Project administration, Resources, Software, Validation, Visualization, Writing – original draft, Writing – review & editing. KV: Conceptualization, Supervision, Writing – review & editing. MS: Conceptualization, Methodology, Supervision, Writing – review & editing.

## Glossary

**Table:**

ADOS	Autism Diagnostic Observation Schedule
ADHD	Attention Deficit Hyperactivity Disorder
AMBIANCE	Atypical Maternal Behavior Instrument for Assessment and Classification
AMHS	Adult Mental Health Service
BPD	Borderline Personality Disorder
CAMHS	Child and Adolescent Mental Health Service
CBT	Cognitive Behavioral Therapy
COMET	Competitive Memory Training
DC:0-5™	Diagnostic Classification of Mental Health and Developmental Disorders of Infancy and Early Childhood
DSM-5	Diagnostic and Statistical Manual of Mental Disorders
EAS	Emotional Availability Scales
EMDR	Eye Movement Desensitization and Reprocessing
FFP	Family Focused Practice
GIT-PD	Guideline Informed Treatment for Personality Disorders
IECMH	Infant and Early Childhood Mental Health
MBT	Mentalization Based Treatment
MIG	Modified Interaction Guidance
PMT	Psych Motor (family-) Therapy
PRT	Pivotal Response Treatment
PTSD	Post Traumatic Stress Disorder
SFT	Schema Focused Therapy
SPSP	Short-term Psychodynamic Supportive Psychotherapy
STEPPS	Systems Training for Emotional Predictability and Problem Solving
TFM	The Family Model

## References

[1] Nicholson J Reupert A Grant A Lees R Maybery D Mordoch E . The policy context and change for families living with parental mental illness. Parental Psychiatr disorder: Distressed parents their families. (2015) 3:354–64. doi: 10.1017/CBO9781107707559.034

[2] Falkov A Goodyear M Hosman CM Biebel K Skogøy BE Kowalenko N . A systems approach to enhance global efforts to implement family-focused mental health interventions. Child Youth Services. (2016) 37:175–93. doi: 10.1080/0145935X.2016.1104104

[3] Leonard RA Linden M Grant A . Family-focused practice for families affected by maternal mental illness and substance misuse in home visiting: A qualitative systematic review. J Family nursing. (2018) 24:128–55. doi: 10.1177/1074840718770612 29683021

[4] Foster K Maybery D Reupert A Gladstone B Grant A Ruud T . Family-focused practice in mental health care: An integrative review. Child Youth Services. (2016) 37:129–55. doi: 10.1080/0145935X.2016.1104048

[5] Bee P Bower P Byford S Churchill R Calam R Stallard P . The clinical effectiveness, cost-effectiveness and acceptability of community-based interventions aimed at improving or maintaining quality of life in children of parents with serious mental illness: A systematic review. Health Technol Assessment. (2014) 18:1–250. doi: 10.3310/hta18080 PMC4780907 24502767

[6] McLaughlin KA Gadermann AM Hwang I Sampson NA Al-Hamzawi A Andrade LH . Parent psychopathology and offspring mental disorders: Results from the WHO World Mental Health Surveys. Br J Psychiatry. (2012) 200:290–9. doi: 10.1192/bjp.bp.111.101253 PMC3317036 22403085

[7] Van Santvoort F Hosman CM Janssens JM Van Doesum KT Reupert A Van Loon LM . The impact of various parental mental disorders on children’s diagnoses: a systematic review. Clin Child Family Psychol Review. (2015) 18:281–99. doi: 10.1007/s10567-015-0191-9 26445808

[8] Campbell TCH Reupert A Sutton K Basu S Davidson G Middeldorp CM . Prevalence of mental illness among parents of children receiving treatment within child and adolescent mental health services (CAMHS): A scoping review. Eur Child Adolesc Psychiatry. (2020) 30:997–1021. doi: 10.1007/s00787-020-01502-x 32133563

[9] Wesseldijk LW Dieleman GC van Steensel FJ Bartels M Hudziak JJ Lindauer RJ . Risk factors for parental psychopathology: a study in families with children or adolescents with psychopathology. Eur Child Adolesc Psychiatry. (2018) 27:1575–84. doi: 10.1007/s00787-018-1156-6 PMC6245117 29644474

[10] Dekelver H Hoekstra A Van Bakel H Marchetta N Van Amelsvoort T . Ten years of infant mental health in the Netherlands: who are the clients? Ment Health Family Med. (2020) 16:974–83.

[11] Sameroff AJ . Ports of Entry and the Dynamics of Mother-Infant Interventions. In: Sameroff AJ McDonough SC Rosenblum K , editors. Treating Parent–infant Relationship Problems: Strategies for Intervention. Guilford Press, New York: Guilford Press (2004). p. 3–28.

[12] Barlow J McMillan A Kirkpatrick S Ghate D Barnes J Smith M . Health-led interventions in the early years to enhance infant and maternal mental health: A review of reviews. Child Adolesc Ment Health. (2010) 15:178–85. doi: 10.1111/j.1475-3588.2010.00570.x 32847203

[13] Brockington I Chandra P Dubowitz H Jones D Moussa S Nakku J . WPA guidance on the protection and promotion of mental health in children of persons with severe mental disorders. World Psychiatry. (2011) 10:93–102. doi: 10.1002/j.2051-5545.2011.tb00023.x 21633678 PMC3104877

[14] O’Shaughnessy R Butterworth R Göpfert M . Assessment and formulation of parenting. In: Reupert A Maybery D Nicholson J Göpfert M Seeman MV , editors. Parental psychiatric disorder: distressed parents and their families, 3rd ed. Cambridge: Cambridge University Press (2015). p. 61–84.

[15] Rouf K Larkin M Lowe G . Making decisions about parental mental health: an exploratory study of community mental health team staff. Child Abuse Review. (2012) 21:173–89. doi: 10.1002/car.1172

[16] Maybery D Foster K Goodyear M Grant A Tungpunkom P Skogoy BE . How can we make the psychiatric workforce more family focused? In: Reupert A Maybery D Nicholson J Göpfert M Seeman MV , editors. Parental Psychiatric Disorder: Distressed Parents and their Families. Cambridge: Cambridge University Press (2015). p. 301–11.

[17] Robson J Gingell K . Improving care for families where children and parents have concurrent mental health problems. Child Adolesc Ment Health. (2012) 17:166–72. doi: 10.1111/j.1475-3588.2011.00630.x 32847271

[18] Maybery D Reupert A . Parental mental illness: A review of barriers and issues for working with families and children. J Psychiatr Ment Health Nursing. (2009) 16:784–91. doi: 10.1111/j.1365-2850.2009.01456.x 19824972

[19] Tuck M Wittkowski A Gregg L . A balancing act: a systematic review and metasynthesis of family-focused practice in adult mental health services. Clin Child Family Psychol Review. (2022) 26:190–211. doi: 10.1007/s10567-022-00418-z PMC9879847 36318397

[20] Tchernegovski P Reupert AE Maybery DJ . How do Australian adult mental health clinicians manage the challenges of working with parental mental illness? A phenomenological study. Child Family Soc Work. (2018) 23:381–9. doi: 10.1111/cfs.12426

[21] Blegen NE Hummelvoll JK Severinsson E . Mothers with mental health problems: a systematic review. Nurs Health Sci. (2010) 12:519–28. doi: 10.1111/j.1442-2018.2010.00550.x 21210933

[22] Van der Ende PC Van Busschbach JT Nicholson J Korevaar EL Van Weeghel J . Strategies for parenting by mothers and fathers with a mental illness. J Psychiatr Ment Health Nursing. (2015) 23:86–97. doi: 10.1111/jpm.12283 26868044

[23] Eaton K Ohan JL Stritzke WGK Corrigan PW . Failing to meet the good parent ideal: self-stigma in parents of children with mental health disorders. J Child Family Stud. (2016) 25:3109–23. doi: 10.1007/s10826-016-0459-9

[24] Huntsman L . Parents with mental health issues: Consequences for children and effectiveness of interventions designed to assist children and their families: Literature review. Ashfield: NSW Department of Community Services (2008). Available at: http://www.community.nsw.gov.au.

[25] Stolper H Doesum KV Steketee M . How to support parents of infants and young children in mental health care: A narrative review. Front Psychol. (2021) 12:745800. doi: 10.3389/fpsyg.2021.745800 34867627 PMC8634941

[26] Stolper H Doesum KV Steketee M . Integrated family approach in mental health care by professionals from adult and child mental health services: A qualitative study. Front Psychol. (2022) 13:781556. doi: 10.3389/fpsyt.2022.781556 PMC9096092 35573344

[27] Stolper H van Doesum K Henselmans P Bijl AL Steketee M . The patient’s voice as a parent in mental health care: A qualitative study. Int J Environ Res Public Health. (2022) 19:13164. doi: 10.3390/ijerph192013164 36293747 PMC9603497

[28] Agorastos A Pervanidou P Chrousos GP Baker DG . Developmental trajectories of early life stress and trauma: a narrative review on neurobiological aspects beyond stress system dysregulation. Front Psychiatry. (2019) 10:118. doi: 10.3389/fpsyt.2019.00118 30914979 PMC6421311

[29] Van den Bergh BR van den Heuvel MI Lahti M Braeken M de Rooij SR Entringer S . Prenatal developmental origins of behavior and mental health: The influence of maternal stress in pregnancy. Neurosci Biobehav Rev. (2020) 117:26–64. doi: 10.1016/j.neubiorev.2017.07.003 28757456

[30] Kowalenko NM Mares SP Newman LK Williams AS Powrie RM Van Doesum KT . Family matters: infants, toddlers and preschoolers of parents affected by mental illness. Med J Aust. (2012) 199:17. doi: 10.5694/mja11.11285 25369842

[31] Fonagy P Bateman AW . Mechanisms of change in mentalization-based treatment of BPD. J Clin Psychol. (2006) 62:411. doi: 10.1002/jclp.20241 16470710

[32] Young JE Klosko JS Weishaar ME . Schema therapy Vol. p. . New York: Guilford (2003). p. 254.

[33] Hutsebaut J Willemsen E Bachrach N Van R . Improving access to and effectiveness of mental health care for personality disorders: the guideline-informed treatment for personality disorders (GIT-PD) initiative in the Netherlands. Borderline Pers Disord Emotional Dysregulation. (2020) 7. doi: 10.1186/s40479-020-00133-7 PMC7416386 32789019

[34] Van Wel B Kockmann I Blum N Pfohl B Black D Heesterman W . STEPPS group treatment for borderline personality disorder in the Netherlands. Ann Clin Psychiatry. (2006) 18:63–7. doi: 10.1080/10401230500464760 16517455

[35] Beck JS . Cognitive behavior therapy: Basics and beyond. 2nd ed. New York: Guilford Press (2011).

[36] Driessen E Cuijpers P De Maat SC Abbass AA De Jonghe F Dekker JJM . The efficacy of short-term psychodynamic psychotherapy for depression: A meta-analysis. Clin Psychol Review. (2010) 30:25–36. doi: 10.1016/j.cpr.2009.08.010 19766369

[37] Shapiro F . Eye movement desensitization and reprocessing (EMDR) therapy: Basic principles, protocols, and procedures. New York: Guilford Publications (2017).

[38] Korrelboom K van der Weele K Gjaltema M Hoogstraten C . Competitive memory training for treating low self-esteem: A pilot study in a routine clinical setting. Behav Therapist. (2009) 23(1):3–8.

[39] Kiep M Spek AA Hoeben L . Mindfulness-based therapy in adults with an autism spectrum disorder: Do treatment effects last? Mindfulness. (2015) 6:637–44. doi: 10.1007/s12671-014-0299-x

[40] Probst M Knapen J Poot G Vancampfort D . Psychomotor therapy and psychiatry: What’s in a name? Open Complement Med J. (2010) 2:105–113. doi: 10.2174/1876391X010020010105

[41] Gonzalez-Dolginko B . Art therapy with adults with autism spectrum disorder. London: Jessica Kingsley Publishers (2019).

[42] James R Sigafoos J Green VA Lancioni GE O’Reilly MF Lang R . Music therapy for individuals with autism spectrum disorder: a systematic review. Rev J Autism Dev Disord. (2015) 2:39–54. doi: 10.1007/s40489-014-0035-4

[43] Nyberg V Hertzmann L . Developing a mentalization-based treatment (MBT) for therapeutic intervention with couples (MBT-CT). J Couple Family Psychoanalysis. (2014) 4:116–35. doi: 10.33212/cfp.v4n2.2014.116

[44] Damen H Veerman JW Vermulst AA van Pagée R Nieuwhoff R Scholte RHJ . Parental empowerment and child behavioral problems during youth care involvement. Child Family Soc Work. (2019) 24:467–76. doi: 10.1111/cfs.12626

[45] Biringen Z Derscheid D Vliegen N Closson L Easterbrooks MA . Emotional Availability (EA): Theoretical background, empirical research using the EA Scales, and clinical applications. Dev Review. (2014) 34:114–67. doi: 10.1016/j.dr.2014.01.002

[46] Lyons-Ruth K Bronfman E Parsons E . Chapter iv. maternal frightened, frightening, or atypical behavior and disorganized infant attachment patterns. Monogr Soc Res Child Dev. (1999) 64:67–96. doi: 10.1111/1540-5834.00034 10597543

[47] Baradon T Broughton C Biseo M Gibbs I James J Joyce A . The Practice of Psychoanalytic Parent-Infant Psychotherapy: Claiming the Baby. United Kingdom: Routledge (2005).

[48] Madigan S Hawkins E Goldberg S Benoit D . Reduction of disrupted caregiver behavior using modified interaction guidance. Infant Ment Health J. (2006) 27:509–27. doi: 10.1002/imhj.20102 28640399

[49] Powell B Cooper G Hoffman K Marvin B . The circle of security intervention: Enhancing attachment in early parent-child relationships. New York: Guilford Publications (2013).

[50] Van Doesum KT Riksen-Walraven JM Hosman CM Hoefnagels C . A randomized controlled trial of a home-visiting intervention aimed at preventing relationship problems in depressed mothers and their infants. Child Dev. (2008) 79:547–61. doi: 10.1111/j.1467-8624.2008.01142.x 18489412

[51] Lovett J . Small wonders: healing childhood trauma with EMDR. New York: Free Press (1999).

[52] Lord C Rutter M Dilavore PC Risi S Gotham K Bishop SL . Autism Diagnostic Observation Schedule – second edition. Los Angeles: Western Psychological Services (2012).

[53] Sameroff AJ . Ports of entry and the dynamics of mother-infant interventions. In: Sameroff AJ McDonough SC Rosenblum KL , editors. Treating parent-infant relationship problems. Strategies of interventions. Guilford Press, London (2004). p. 1–43.

[54] Hosman CMH van Doesum KTM van Santvoort F . Prevention of emotional problems and psychiatric risks in children of parents with a mental illness in the Netherlands: I. The scientific basis to a comprehensive approach. Aust e-Journal Advancement Ment Health. (2009) 8:250–63. doi: 10.5172/jamh.8.3.250

[55] Felitti VJ Anda RF Nordenberg D Williamson DF Spitz AM Edwards V . Relationship of childhood abuse and household dysfunction to many of the leading causes of death in adults. Am J Prev Med. (1998) 14:245–58. doi: 10.1016/s0749-3797(98)00017-8 9635069

[56] Everts S van Amelsvoort T Leijdesdorff S . Mandatory check for COPMI in adult mental healthcare services in the Netherlands—A quantitative and qualitative evaluation. Front Psychiatry. (2022) 13:807251. doi: 10.3389/fpsyt.2022.807251 35370848 PMC8971626

[57] Maybery D Goodyear M Reupert AE Grant A . Worker, workplace or families: What influences family focused practices in adult mental health? J Psychiatr Ment Health Nursing. (2016) 23:163–71. doi: 10.1111/jpm.12294 27170070

[58] Falkov A Grant A Hoadley B Donaghy M Weimand BM . The family model: a brief intervention for clinicians in adult mental health services working with parents experiencing mental health problems. Aust New Z J Psychiatry. (2020) 54:449–52. doi: 10.1177/0004867420913614 32306745

[59] Falkov A . The Family Model Handbook: An integrated approach to supporting mentally ill parents and their children. Pavilion, Brighton: Pavilion (2012).

